# Supplementary material for: MicroRNAs Expression Profile in MN1-Altered Astroblastoma
Source: Biomedicines. 2025 Jan 6;13(1):112. doi: 10.3390/biomedicines13010112 (PMC11762140; doi:10.3390/biomedicines13010112)
Supplement: Supplementary file 1 [file biomedicines-13-00112-s001.zip › Figure S2.pptx]

## Slide 1
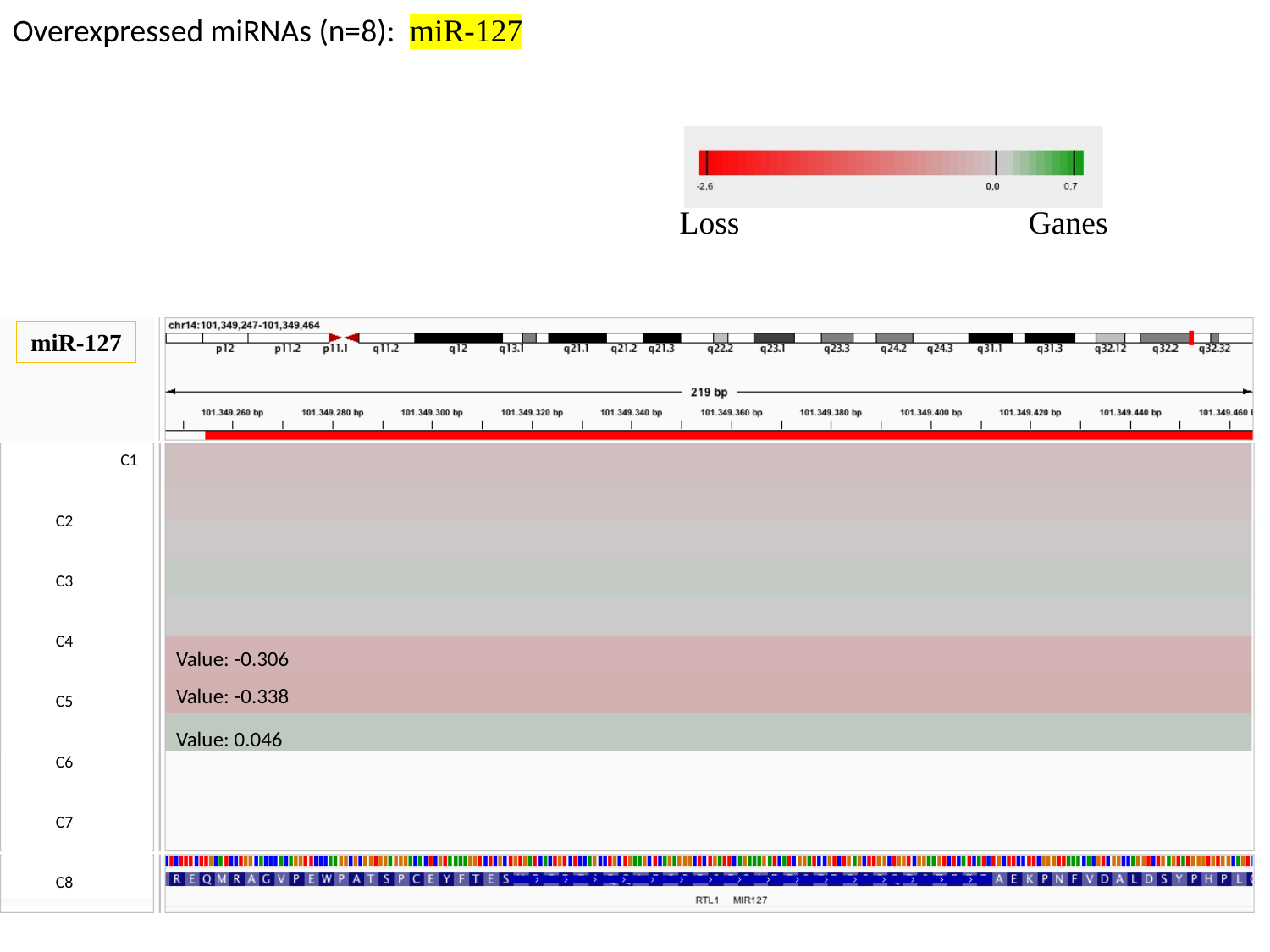

Overexpressed miRNAs (n=8): miR-127
Loss Ganes
Value: -0.306
Value: -0.338
Value: 0.046
 C1
	 C2
	 C3
	 C4
	 C5
	 C6
	 C7
	 C8
miR-127

## Slide 2
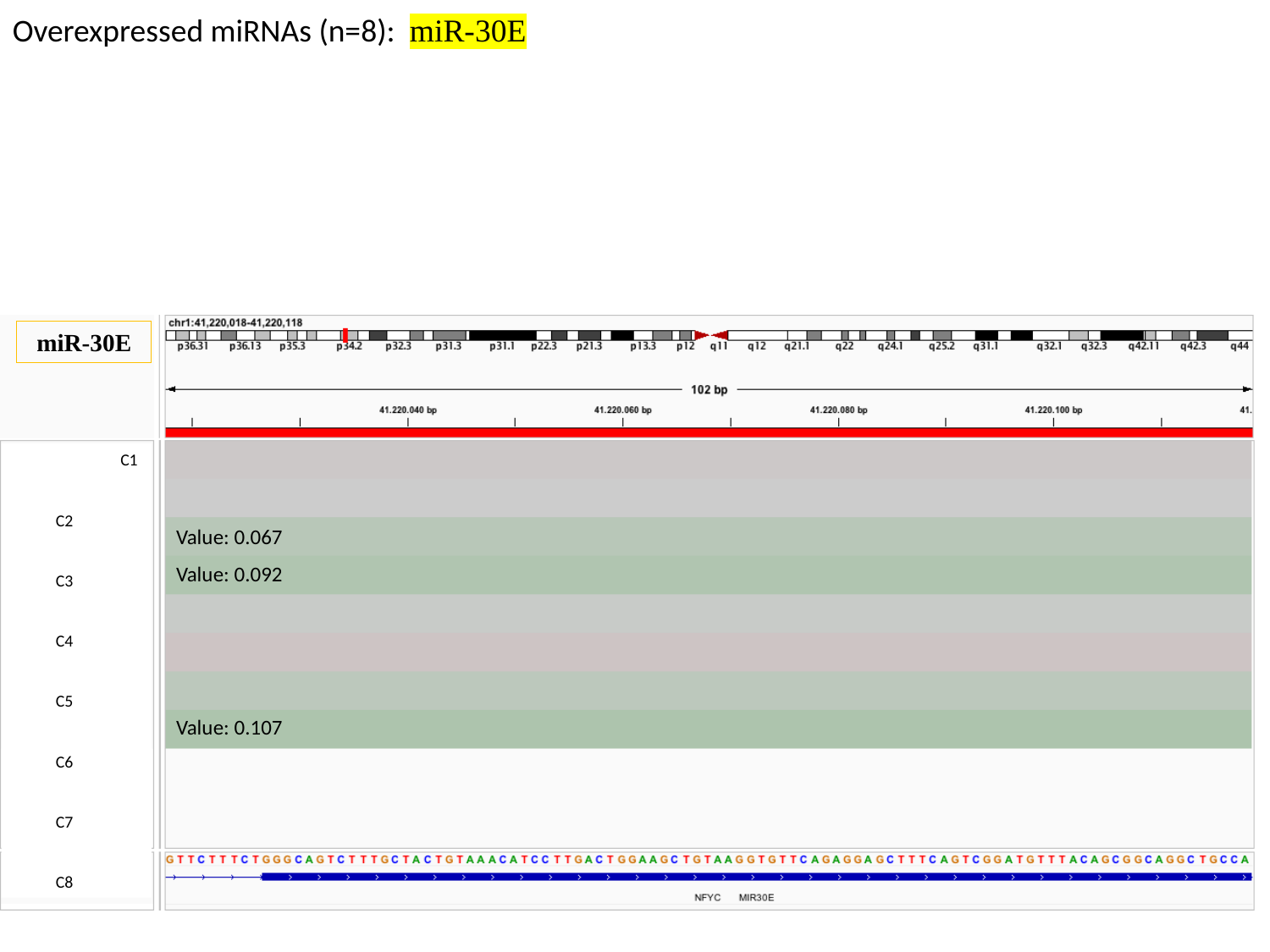

Overexpressed miRNAs (n=8): miR-30E
Value: 0.067
Value: 0.092
Value: 0.107
 C1
	 C2
	 C3
	 C4
	 C5
	 C6
	 C7
	 C8
miR-30E

## Slide 3
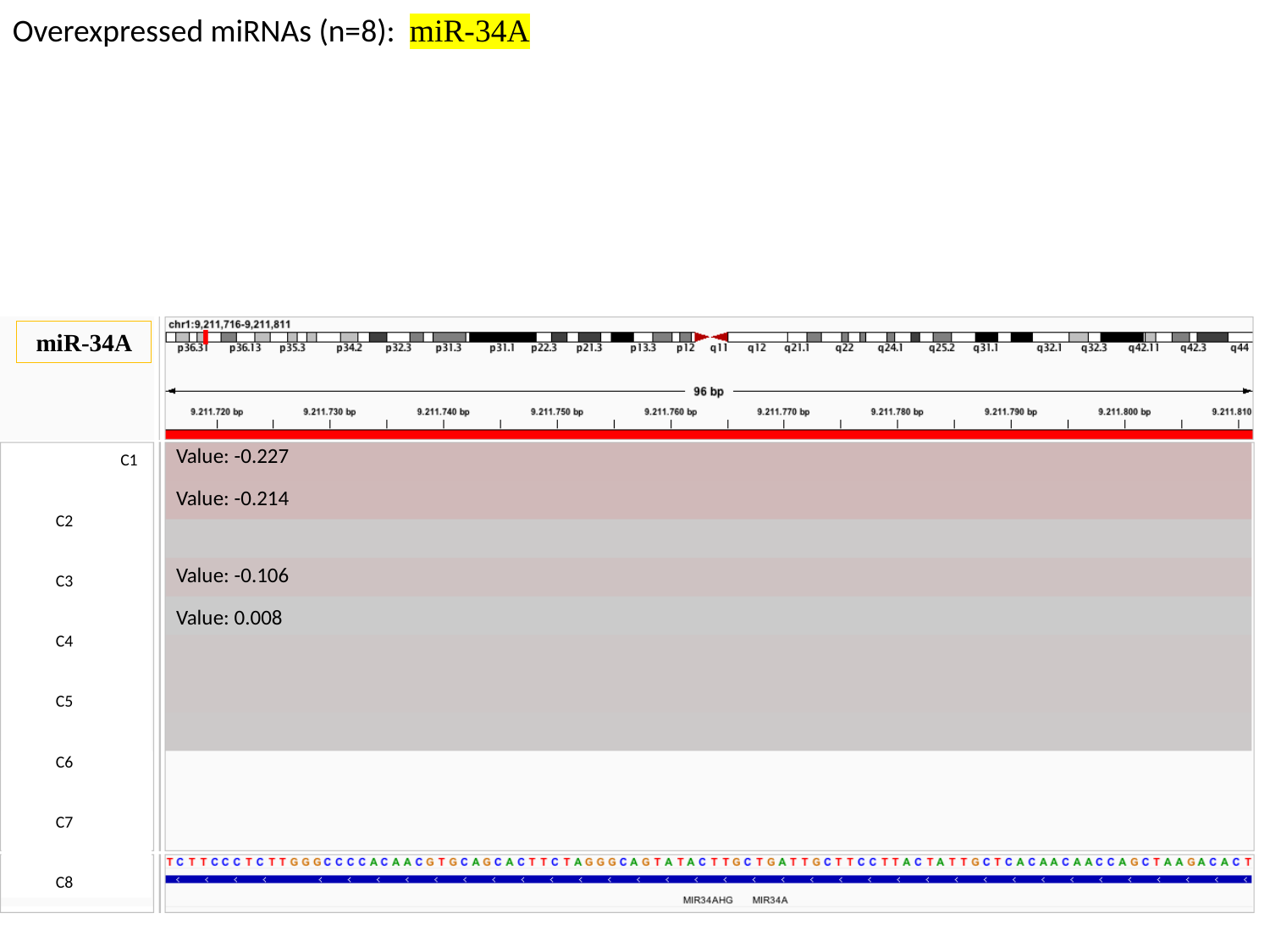

Overexpressed miRNAs (n=8): miR-34A
Value: -0.227
Value: -0.214
Value: -0.106
Value: 0.008
 C1
	 C2
	 C3
	 C4
	 C5
	 C6
	 C7
	 C8
miR-34A

## Slide 4
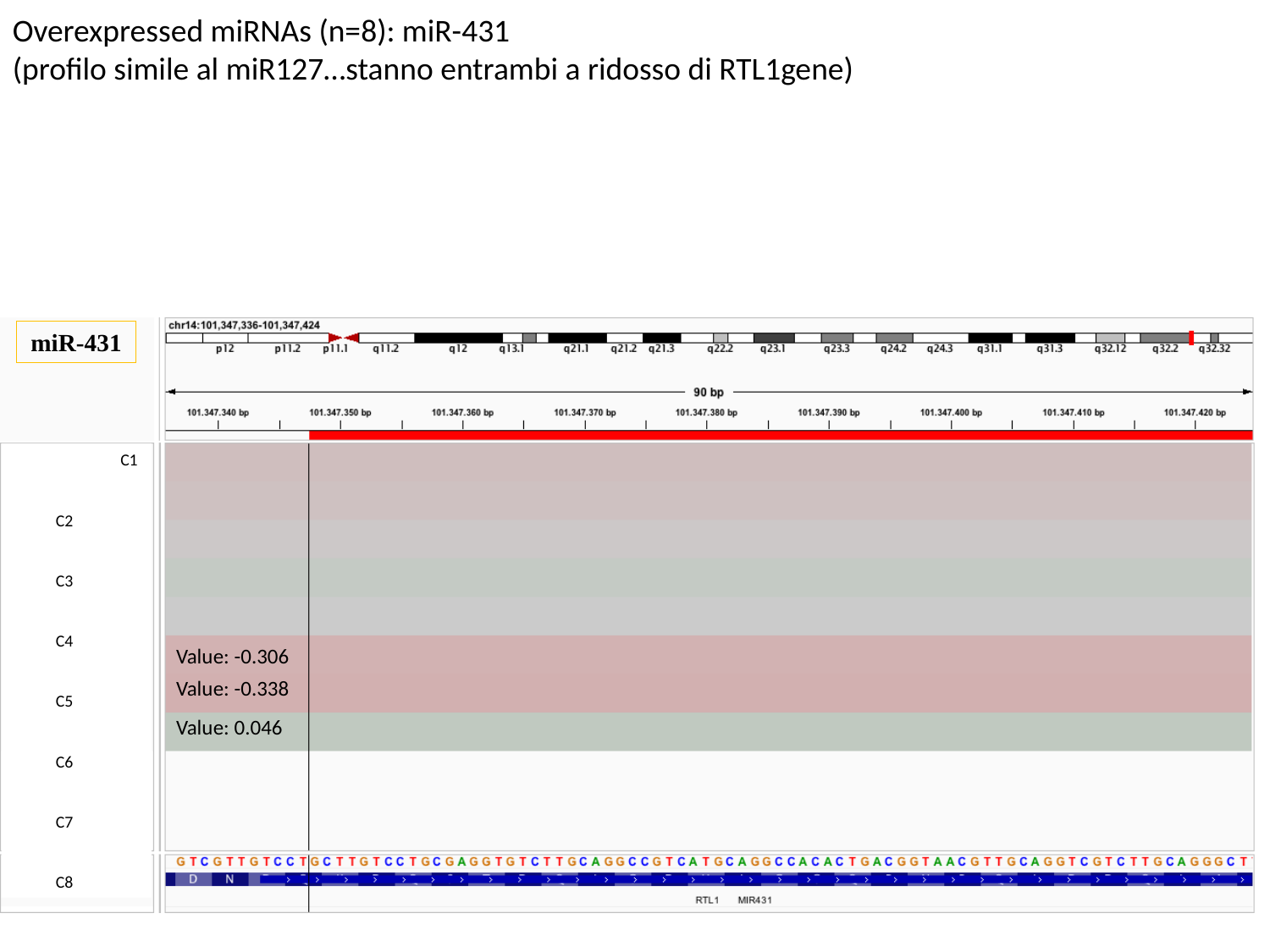

Overexpressed miRNAs (n=8): miR-431
(profilo simile al miR127…stanno entrambi a ridosso di RTL1gene)
Value: -0.306
Value: -0.338
Value: 0.046
 C1
	 C2
	 C3
	 C4
	 C5
	 C6
	 C7
	 C8
miR-431

## Slide 5
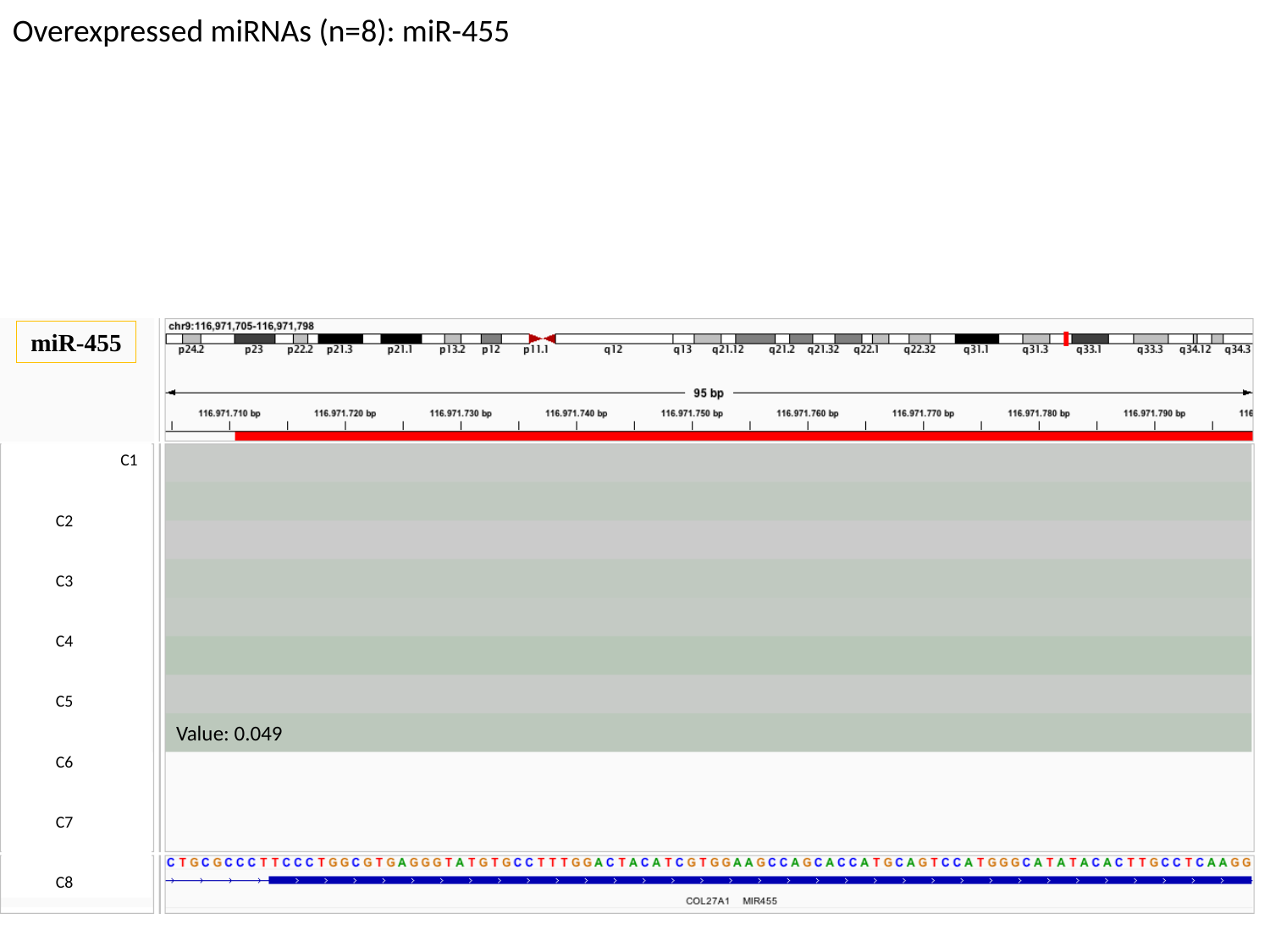

Overexpressed miRNAs (n=8): miR-455
Value: 0.049
 C1
	 C2
	 C3
	 C4
	 C5
	 C6
	 C7
	 C8
miR-455

## Slide 6
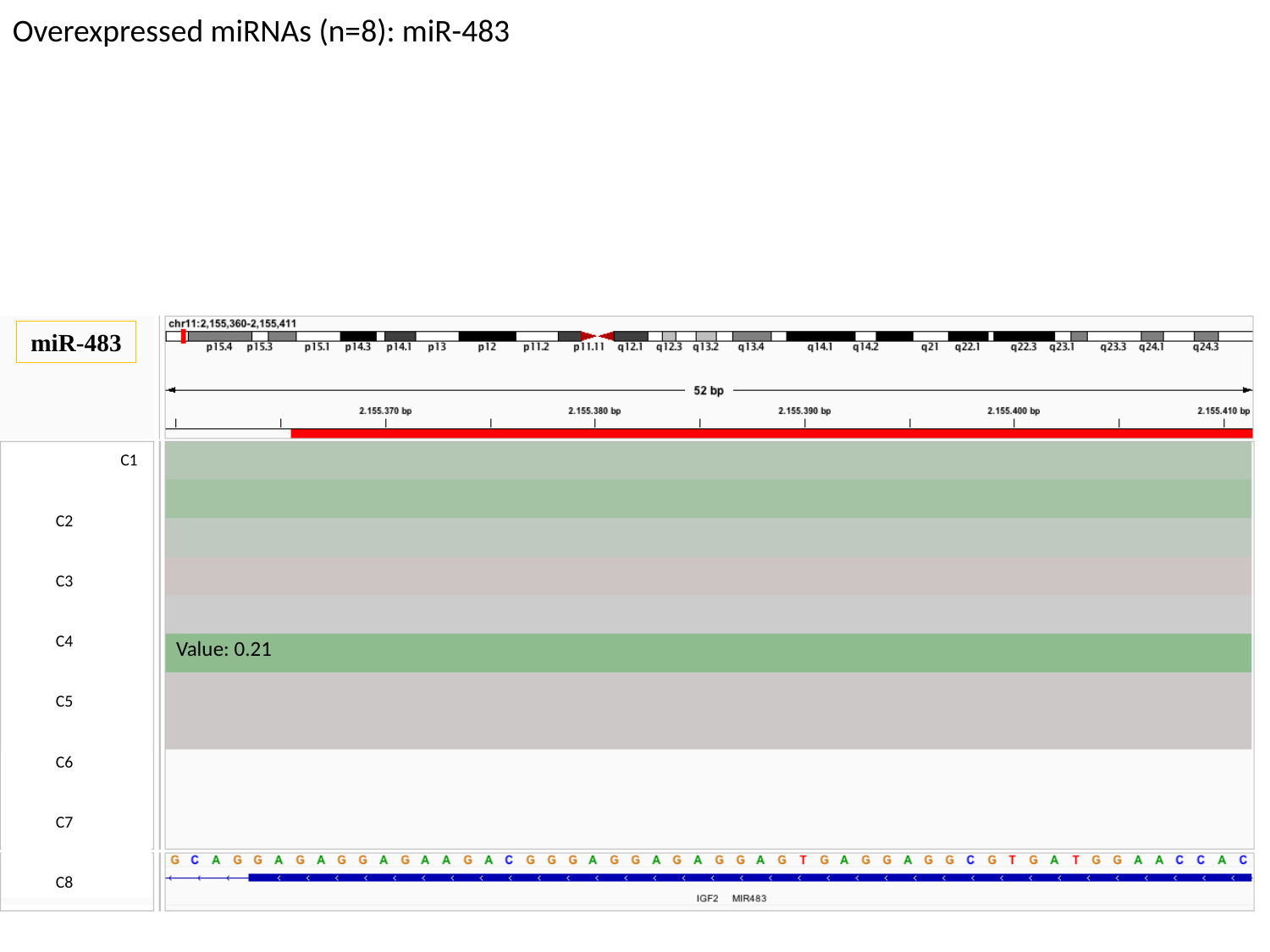

Overexpressed miRNAs (n=8): miR-483
Value: 0.21
 C1
	 C2
	 C3
	 C4
	 C5
	 C6
	 C7
	 C8
miR-483

## Slide 7
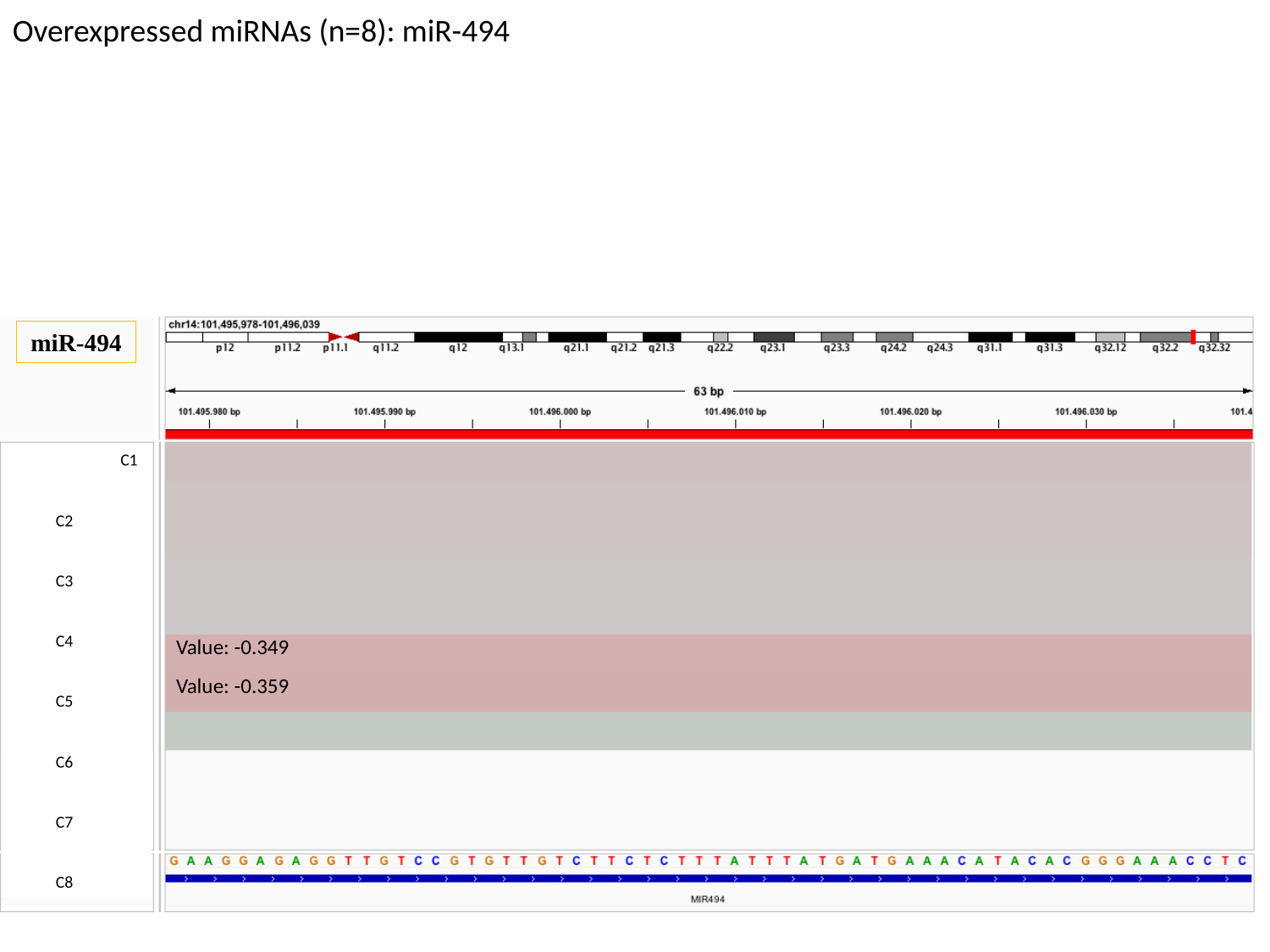

Overexpressed miRNAs (n=8): miR-494
Value: -0.349
Value: -0.359
 C1
	 C2
	 C3
	 C4
	 C5
	 C6
	 C7
	 C8
miR-494

## Slide 8
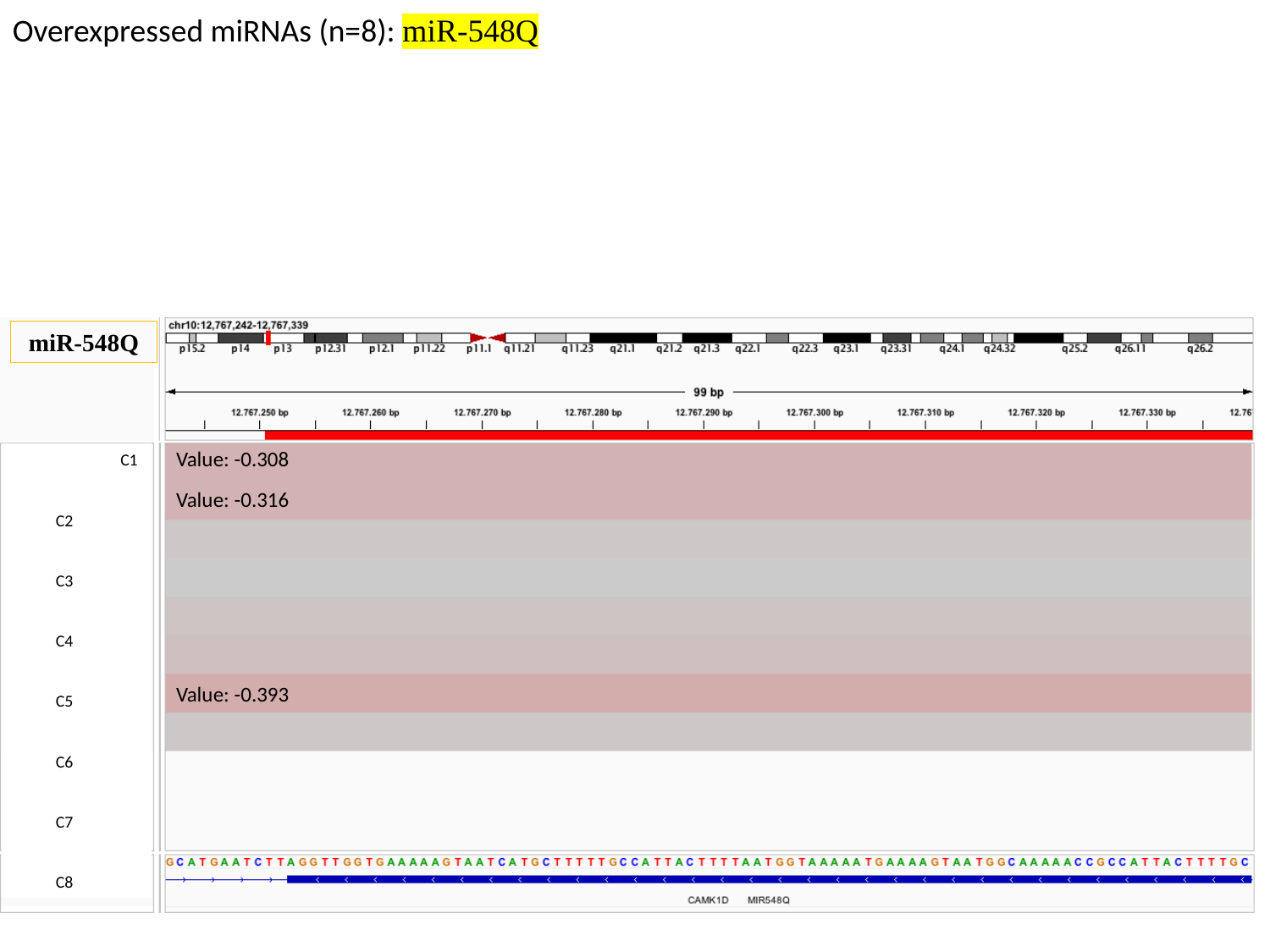

Overexpressed miRNAs (n=8): miR-548Q
Value: -0.308
Value: -0.316
Value: -0.393
 C1
	 C2
	 C3
	 C4
	 C5
	 C6
	 C7
	 C8
miR-548Q

## Slide 9
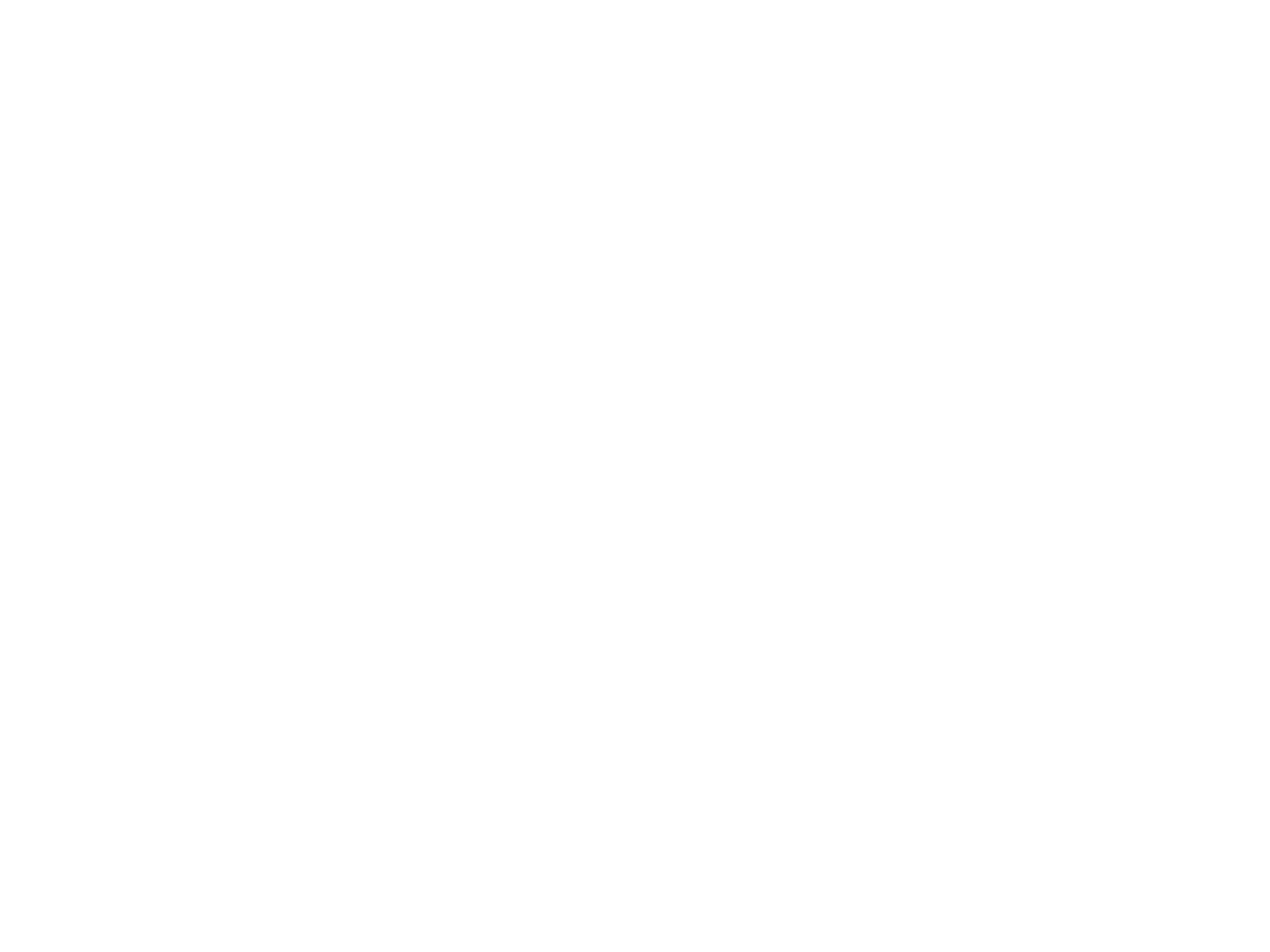

## Slide 10
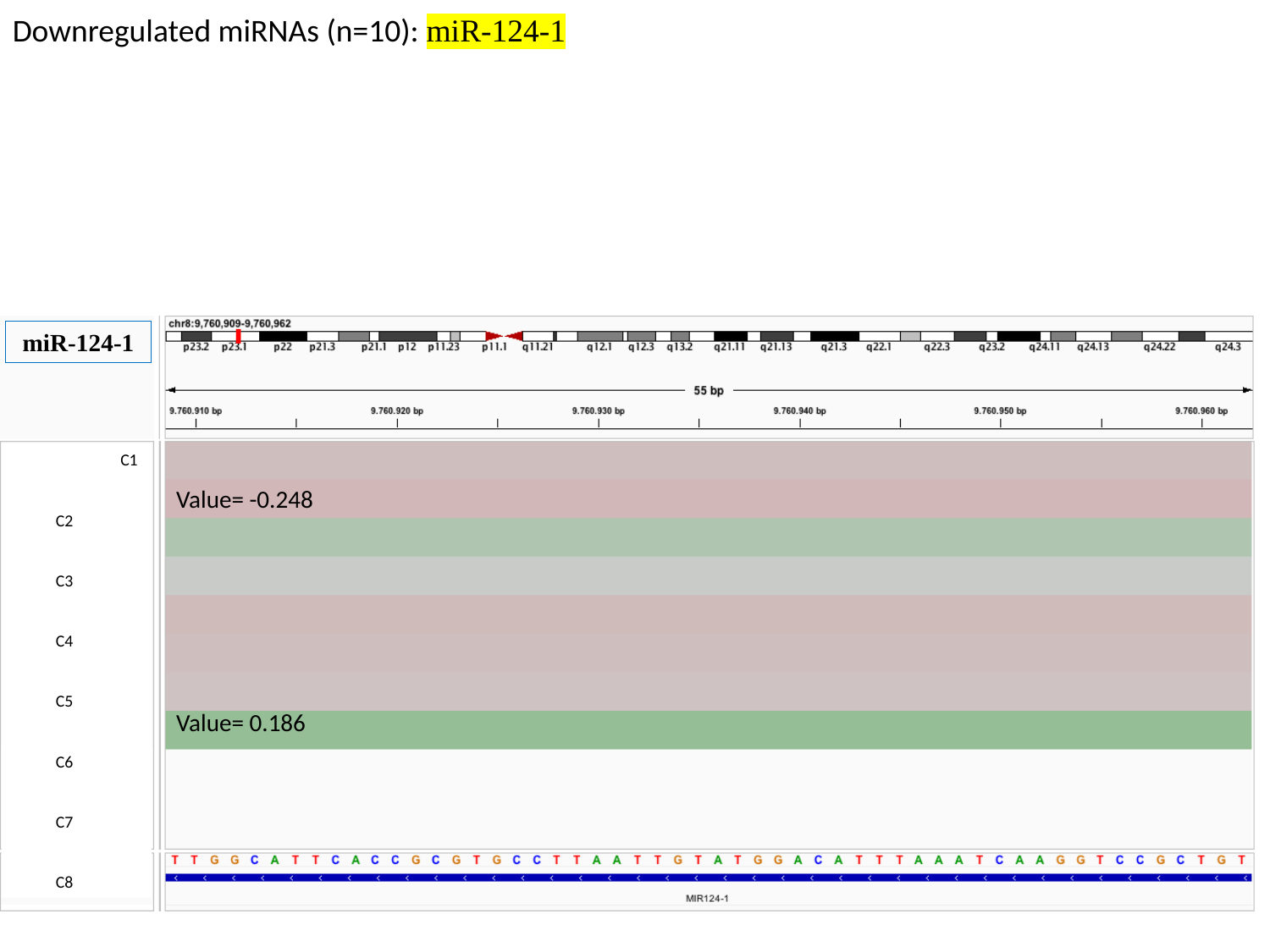

Downregulated miRNAs (n=10): miR-124-1
Value= -0.248
Value= 0.186
 C1
	 C2
	 C3
	 C4
	 C5
	 C6
	 C7
	 C8
miR-124-1

## Slide 11
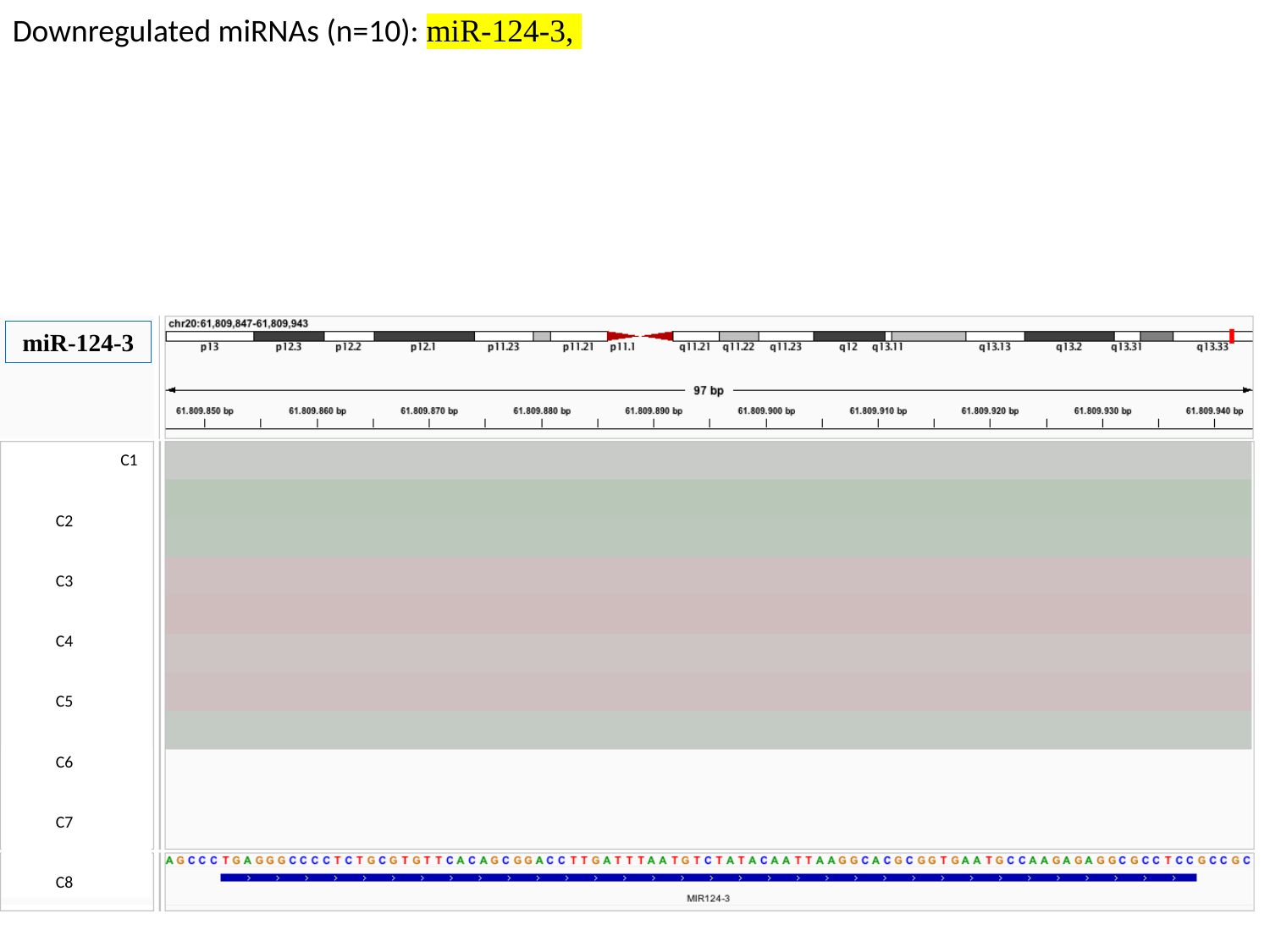

Downregulated miRNAs (n=10): miR-124-3,
 C1
	 C2
	 C3
	 C4
	 C5
	 C6
	 C7
	 C8
miR-124-3

## Slide 12
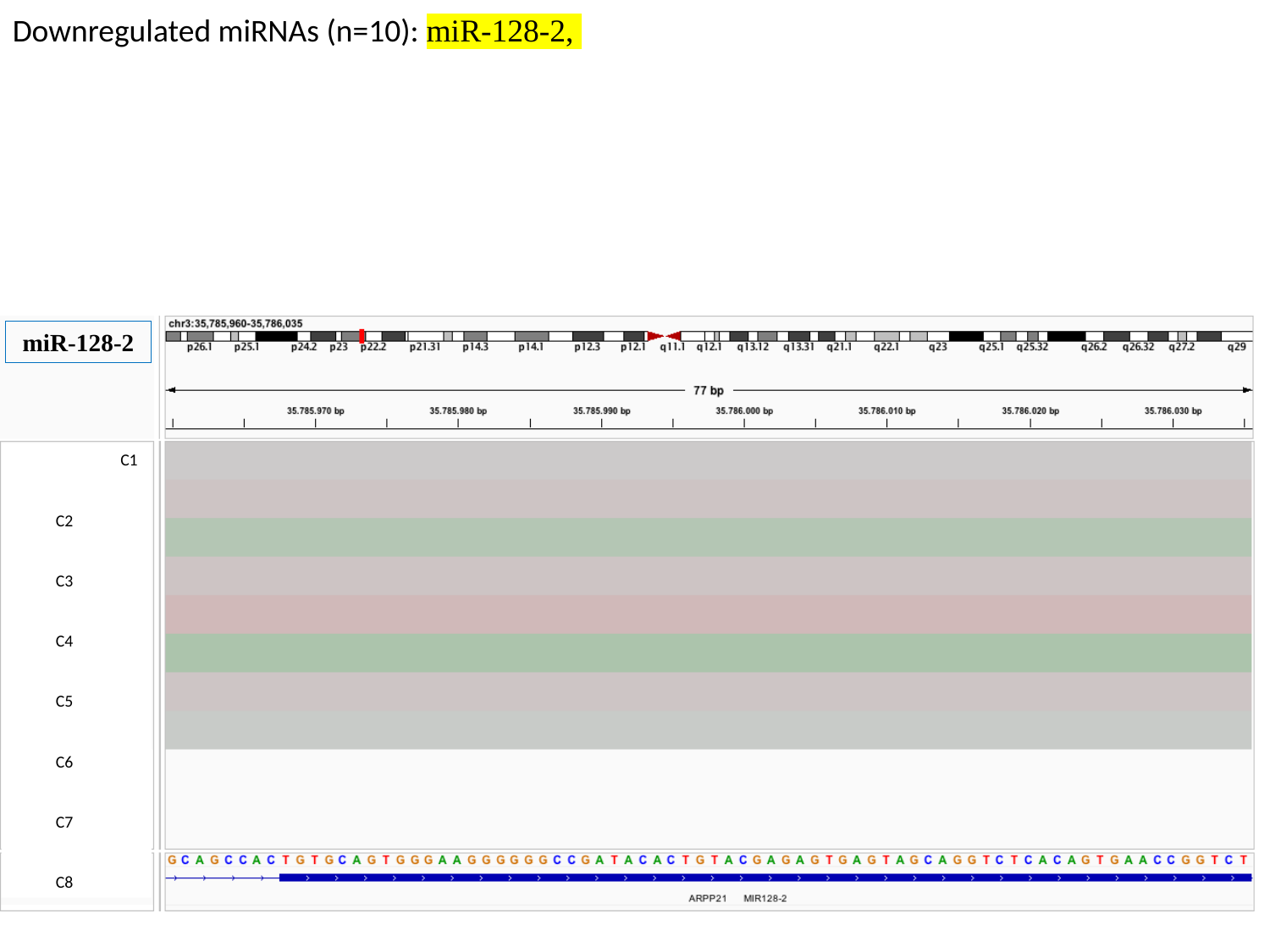

Downregulated miRNAs (n=10): miR-128-2,
 C1
	 C2
	 C3
	 C4
	 C5
	 C6
	 C7
	 C8
miR-128-2

## Slide 13
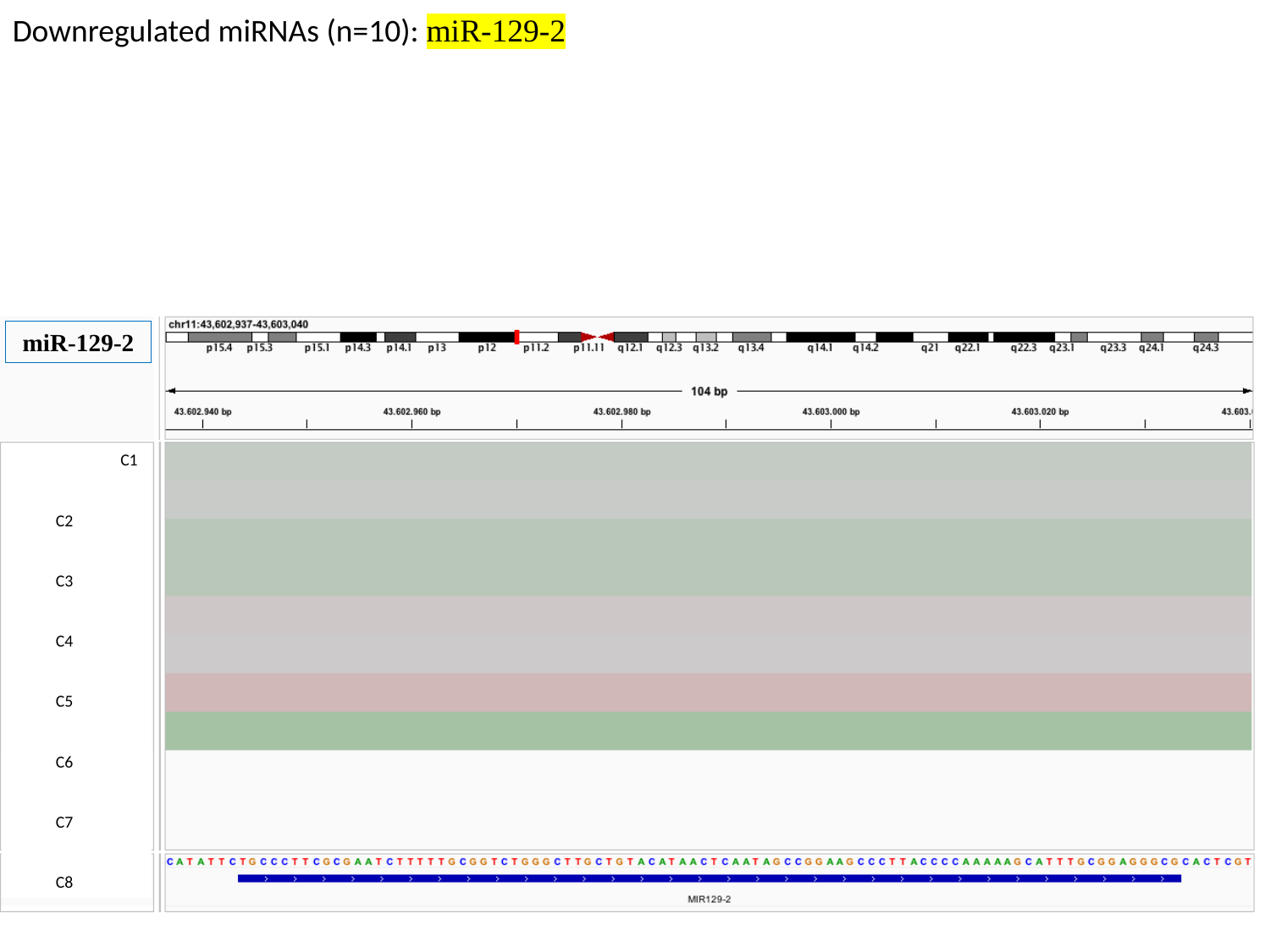

Downregulated miRNAs (n=10): miR-129-2
 C1
	 C2
	 C3
	 C4
	 C5
	 C6
	 C7
	 C8
miR-129-2

## Slide 14
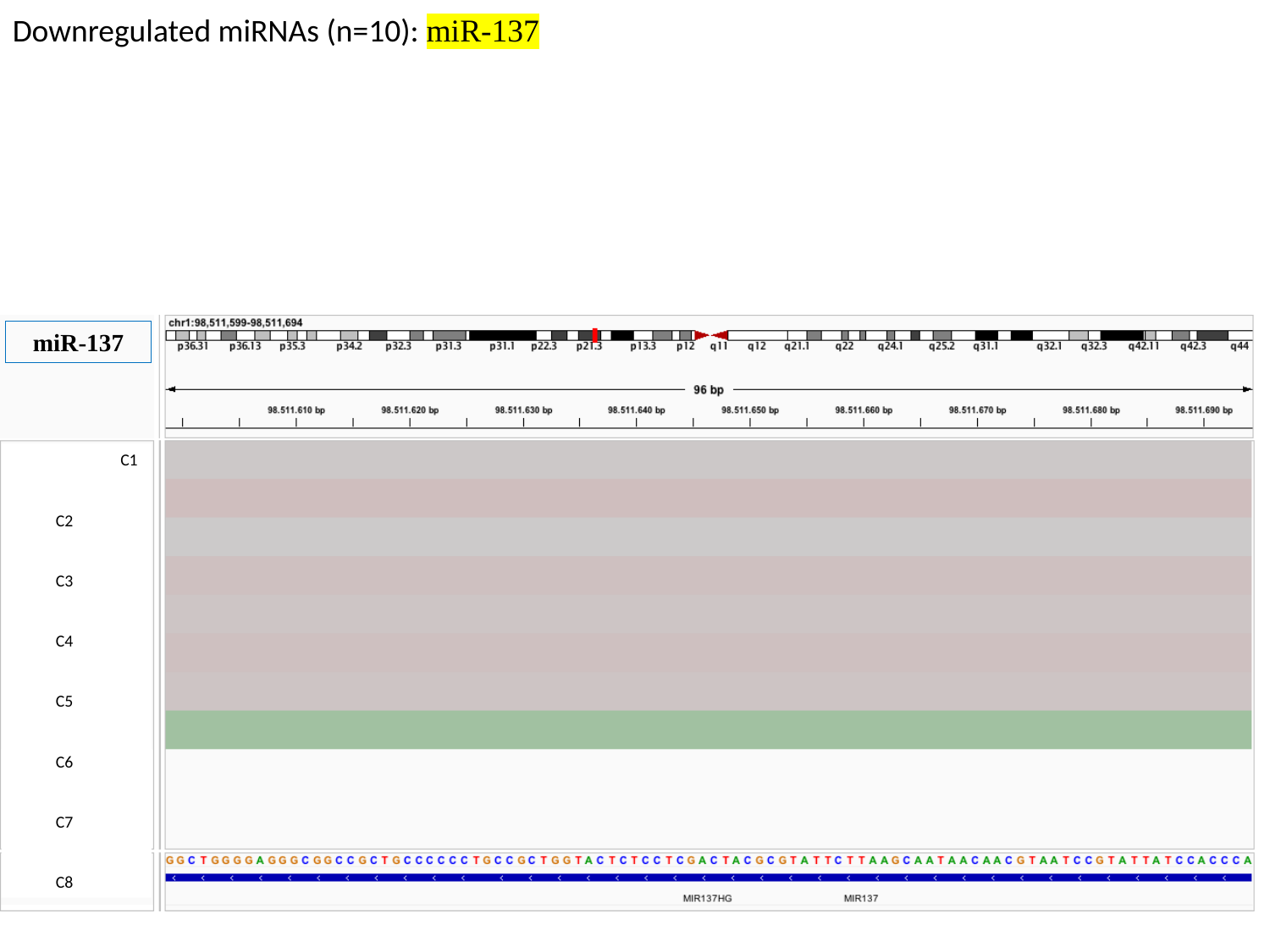

Downregulated miRNAs (n=10): miR-137
 C1
	 C2
	 C3
	 C4
	 C5
	 C6
	 C7
	 C8
miR-137

## Slide 15
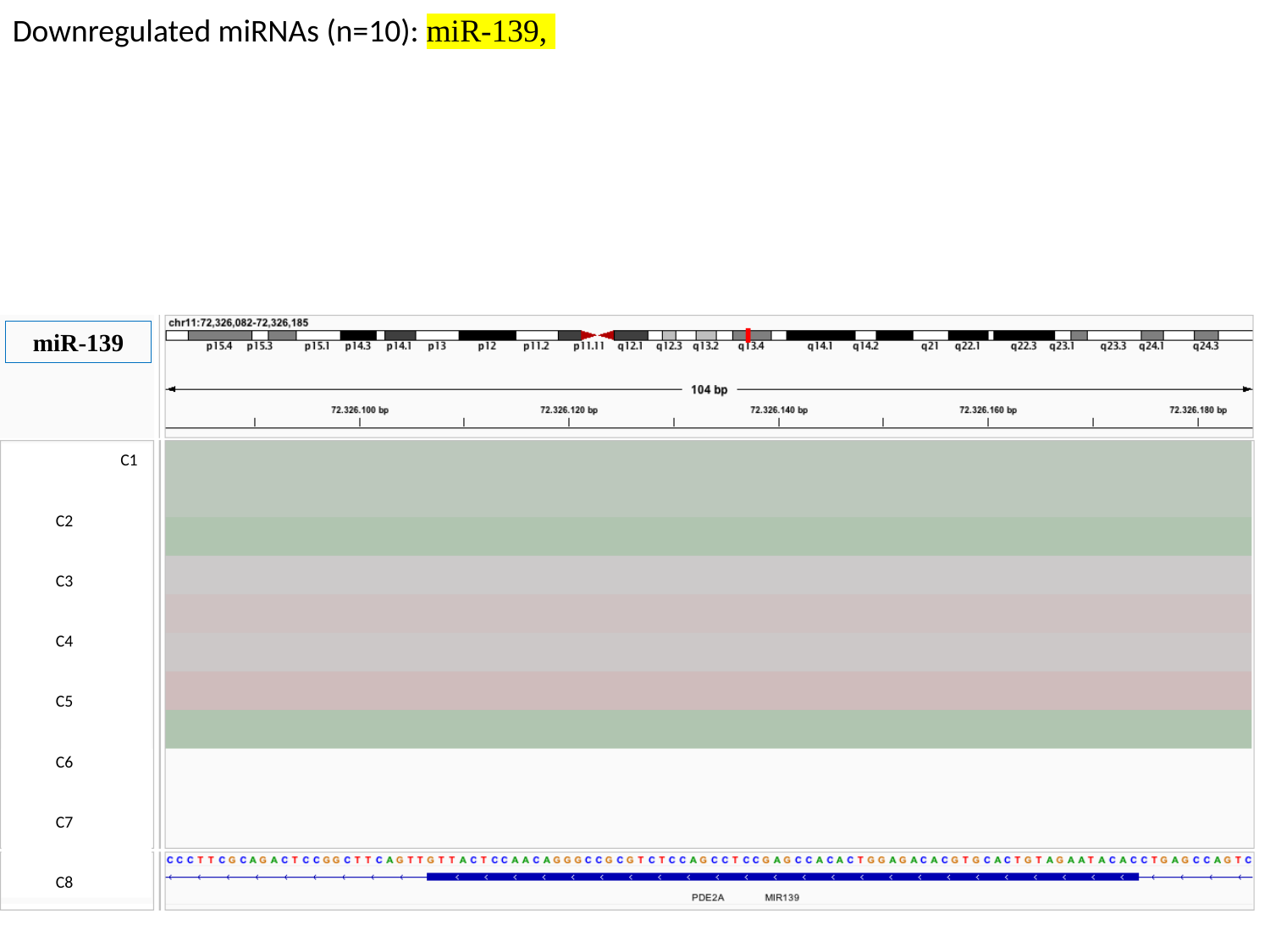

Downregulated miRNAs (n=10): miR-139,
 C1
	 C2
	 C3
	 C4
	 C5
	 C6
	 C7
	 C8
miR-139

## Slide 16
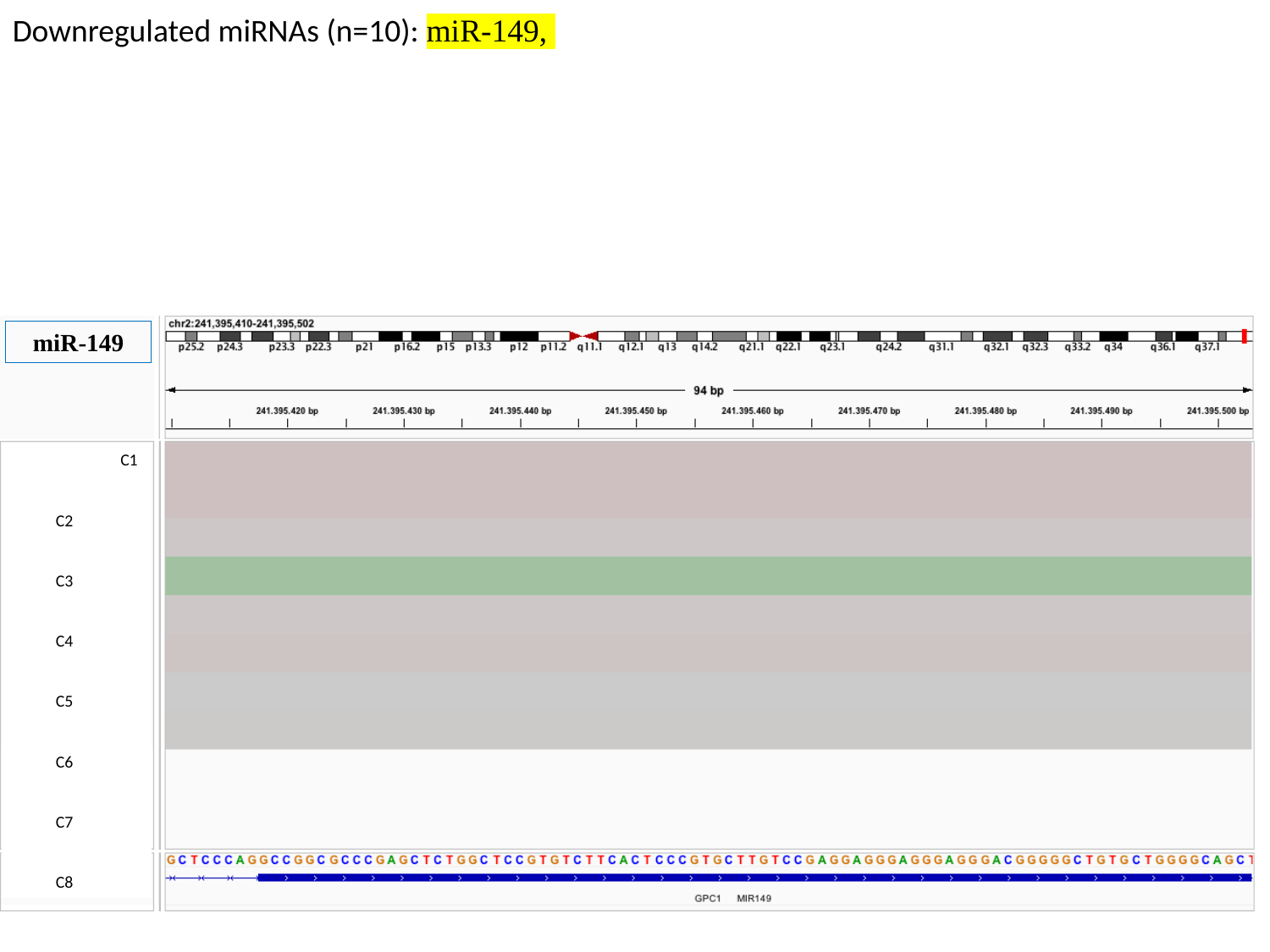

Downregulated miRNAs (n=10): miR-149,
 C1
	 C2
	 C3
	 C4
	 C5
	 C6
	 C7
	 C8
miR-149

## Slide 17
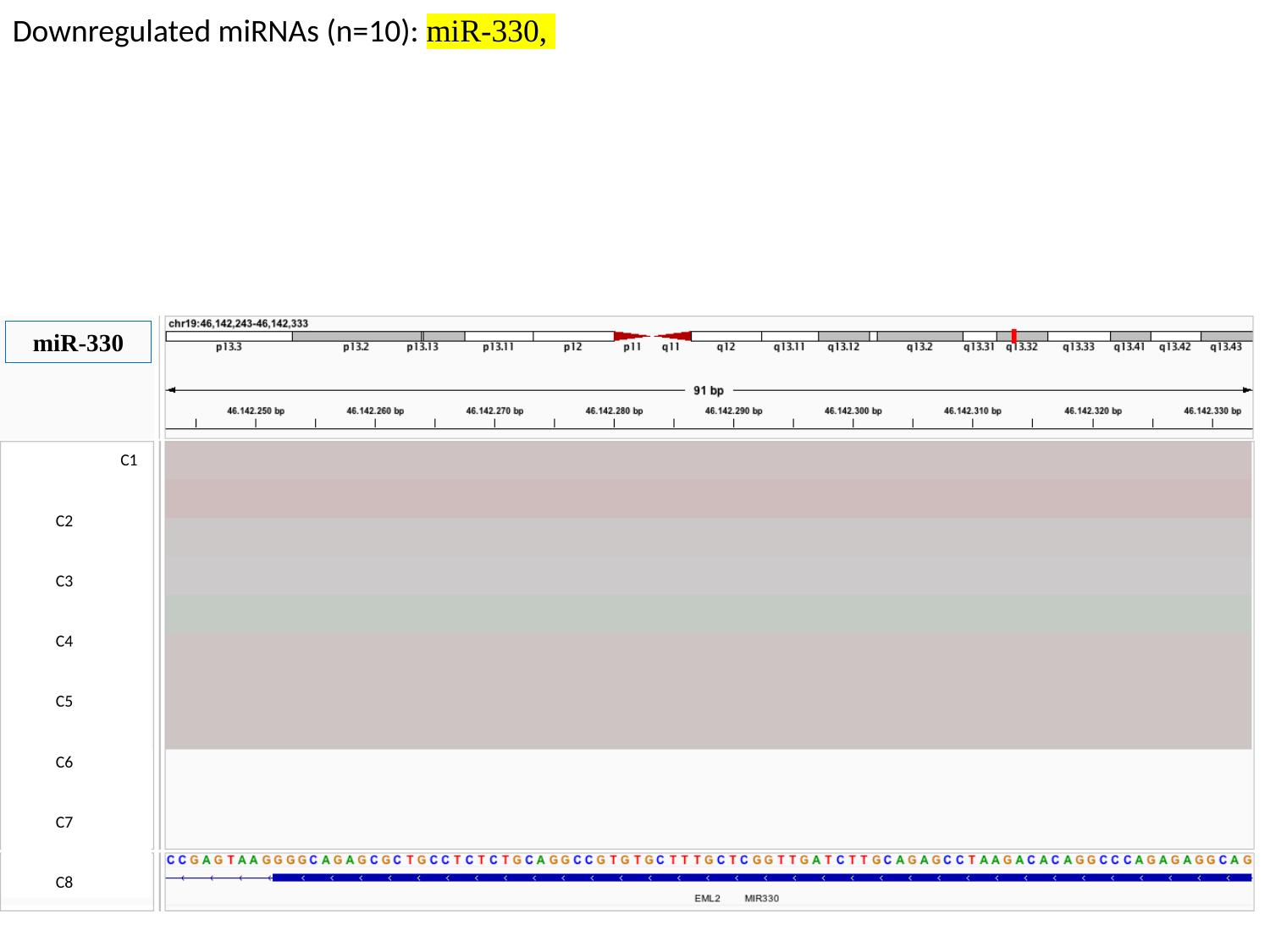

Downregulated miRNAs (n=10): miR-330,
 C1
	 C2
	 C3
	 C4
	 C5
	 C6
	 C7
	 C8
miR-330

## Slide 18
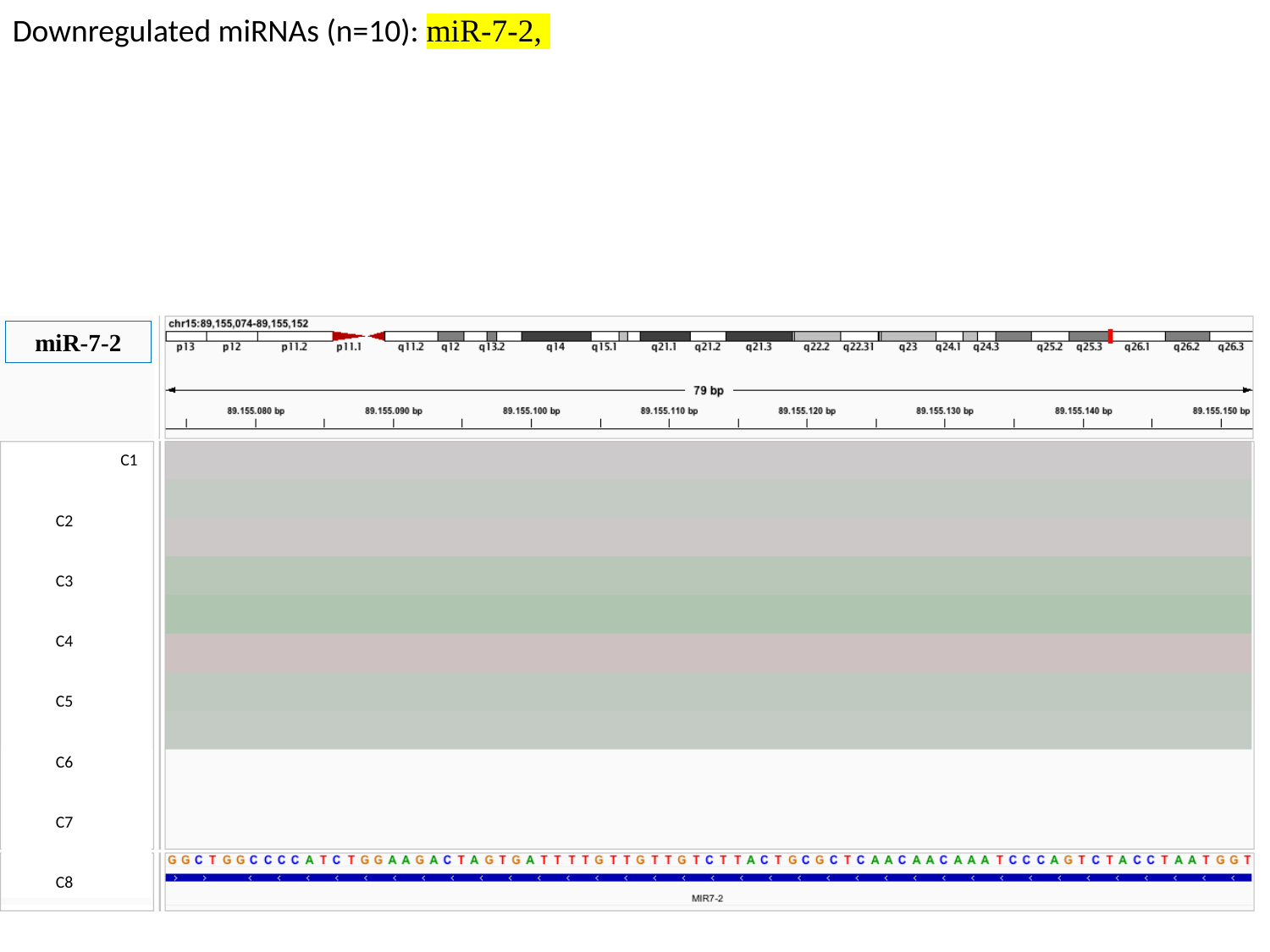

Downregulated miRNAs (n=10): miR-7-2,
 C1
	 C2
	 C3
	 C4
	 C5
	 C6
	 C7
	 C8
miR-7-2

## Slide 19
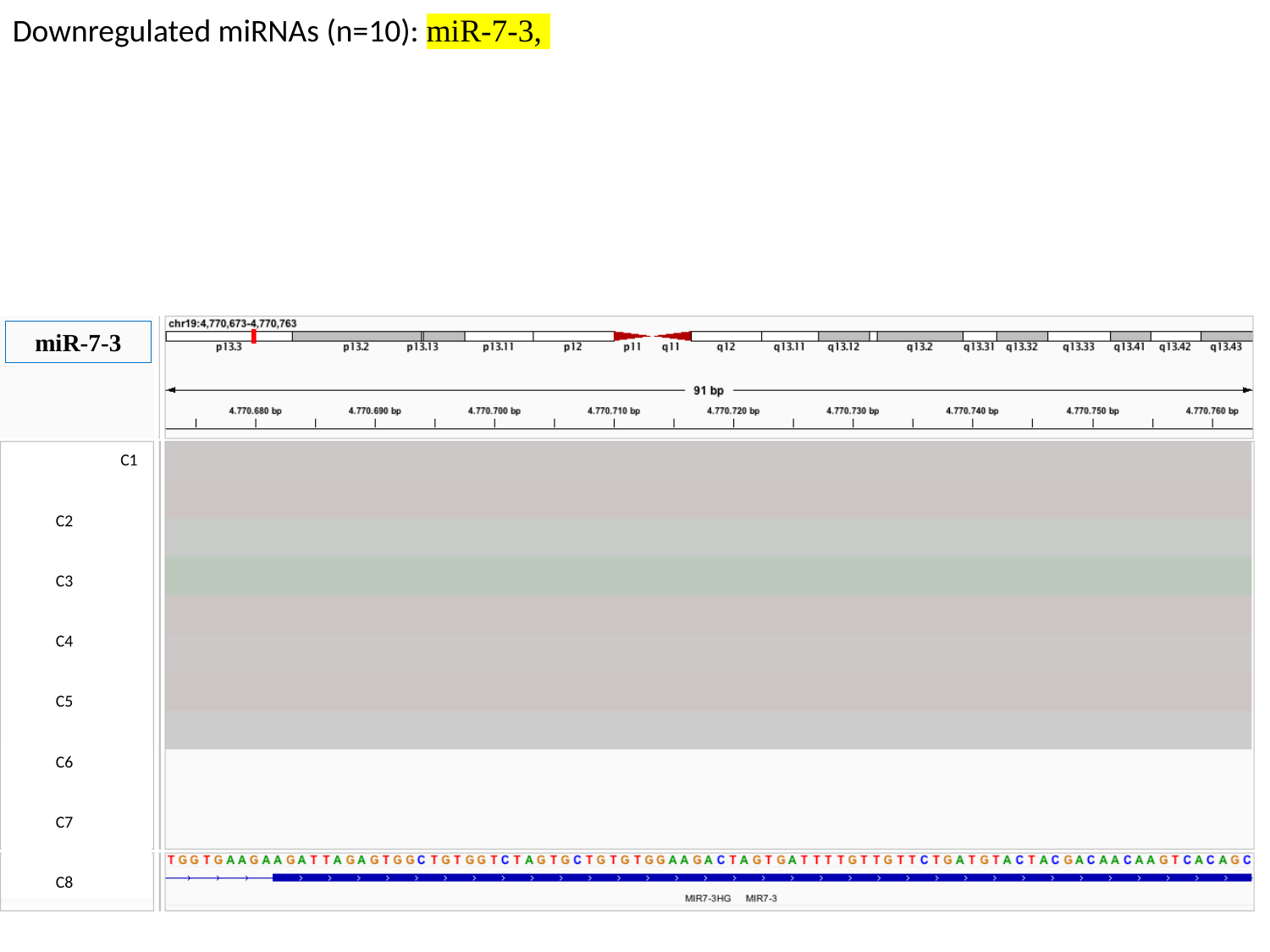

Downregulated miRNAs (n=10): miR-7-3,
 C1
	 C2
	 C3
	 C4
	 C5
	 C6
	 C7
	 C8
miR-7-3

## Slide 20
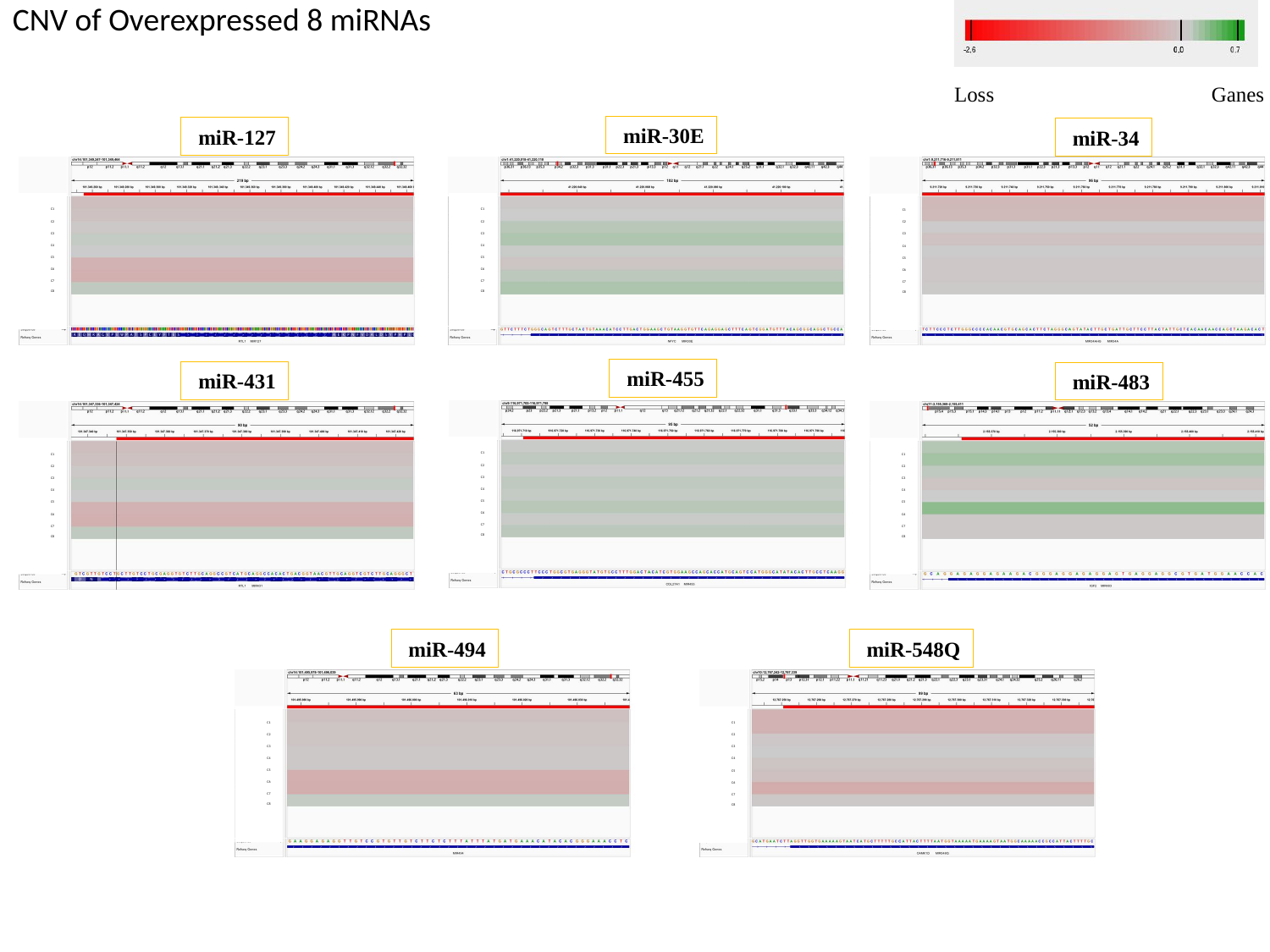

Loss Ganes
CNV of Overexpressed 8 miRNAs
 miR-30E
 miR-127
 miR-34
 C1
C2
C3
C4
C5
C6
C7
C8
 C1
C2
C3
C4
C5
C6
C7
C8
 C1
C2
C3
C4
C5
C6
C7
C8
 C1
C2
C3
C4
C5
C6
C7
C8
 miR-455
 miR-431
 miR-483
 C1
C2
C3
C4
C5
C6
C7
C8
 C1
C2
C3
C4
C5
C6
C7
C8
 C1
C2
C3
C4
C5
C6
C7
C8
 miR-494
 miR-548Q
 C1
C2
C3
C4
C5
C6
C7
C8
 C1
C2
C3
C4
C5
C6
C7
C8

## Slide 21
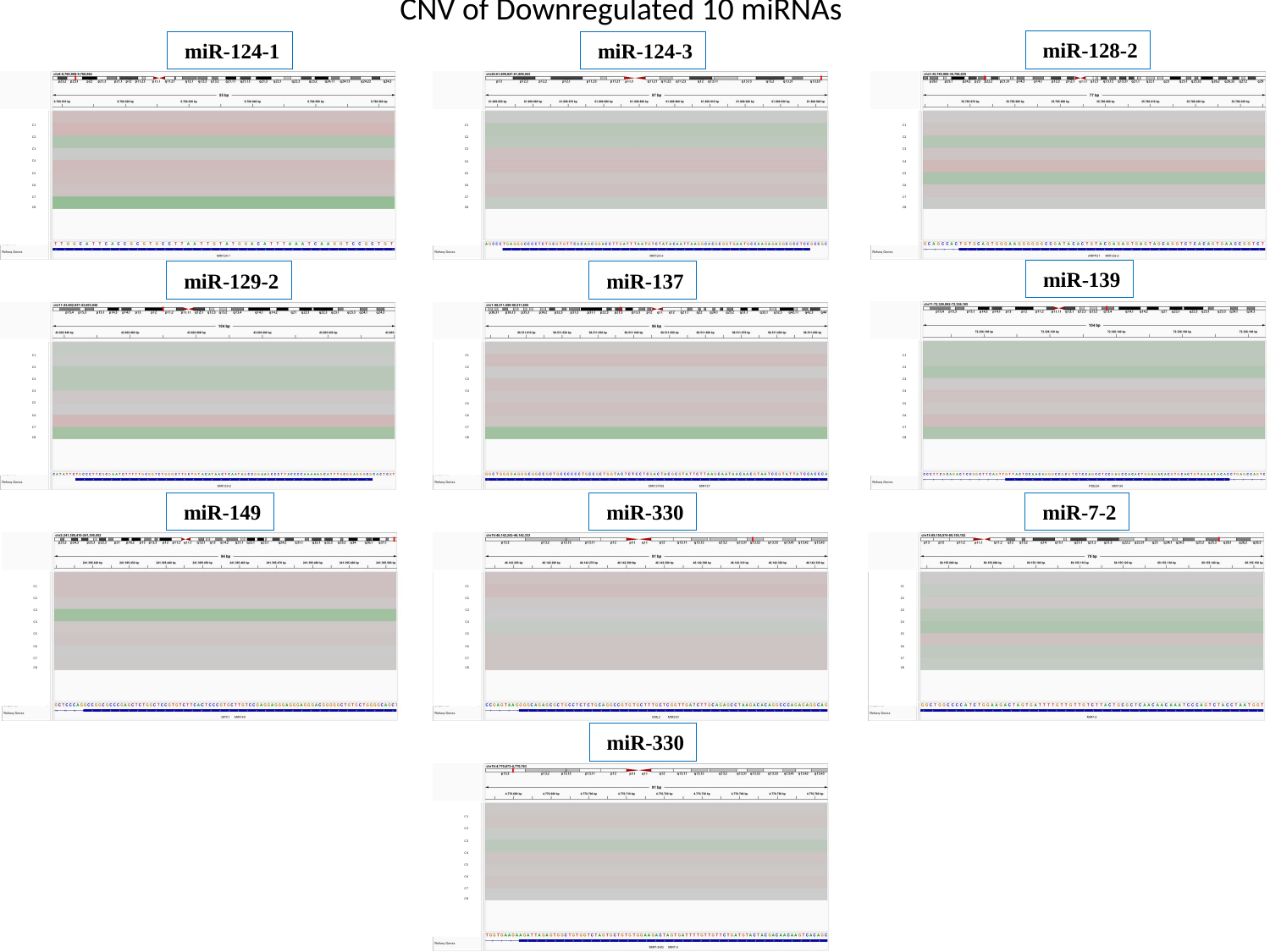

CNV of Downregulated 10 miRNAs
 miR-128-2
 miR-124-1
 miR-124-3
 C1
C2
C3
C4
C5
C6
C7
C8
 C1
C2
C3
C4
C5
C6
C7
C8
 C1
C2
C3
C4
C5
C6
C7
C8
 miR-139
 miR-129-2
 miR-137
 C1
C2
C3
C4
C5
C6
C7
C8
 C1
C2
C3
C4
C5
C6
C7
C8
 C1
C2
C3
C4
C5
C6
C7
C8
 miR-149
 miR-330
 miR-7-2
 C1
C2
C3
C4
C5
C6
C7
C8
 C1
C2
C3
C4
C5
C6
C7
C8
 C1
C2
C3
C4
C5
C6
C7
C8
 miR-330
 C1
C2
C3
C4
C5
C6
C7
C8
