# Supplementary figures and images for: MicroRNAs Expression Profile in MN1-Altered Astroblastoma
Source: Biomedicines. 2025 Jan 6;13(1):112. doi: 10.3390/biomedicines13010112 (PMC11762140; doi:10.3390/biomedicines13010112)

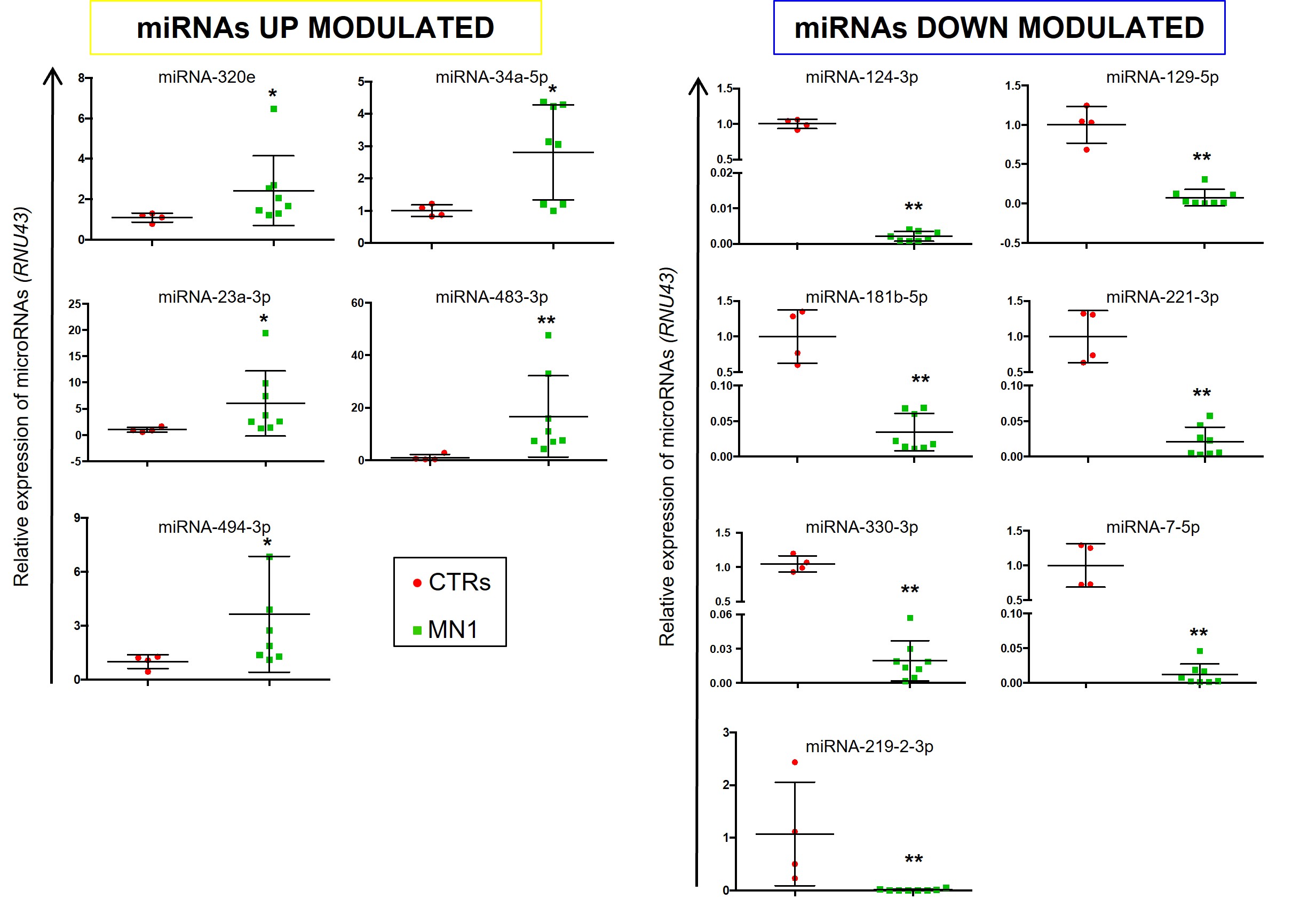

Supplement: Supplementary file 1 [file biomedicines-13-00112-s001.zip › Figure S1.jpg]

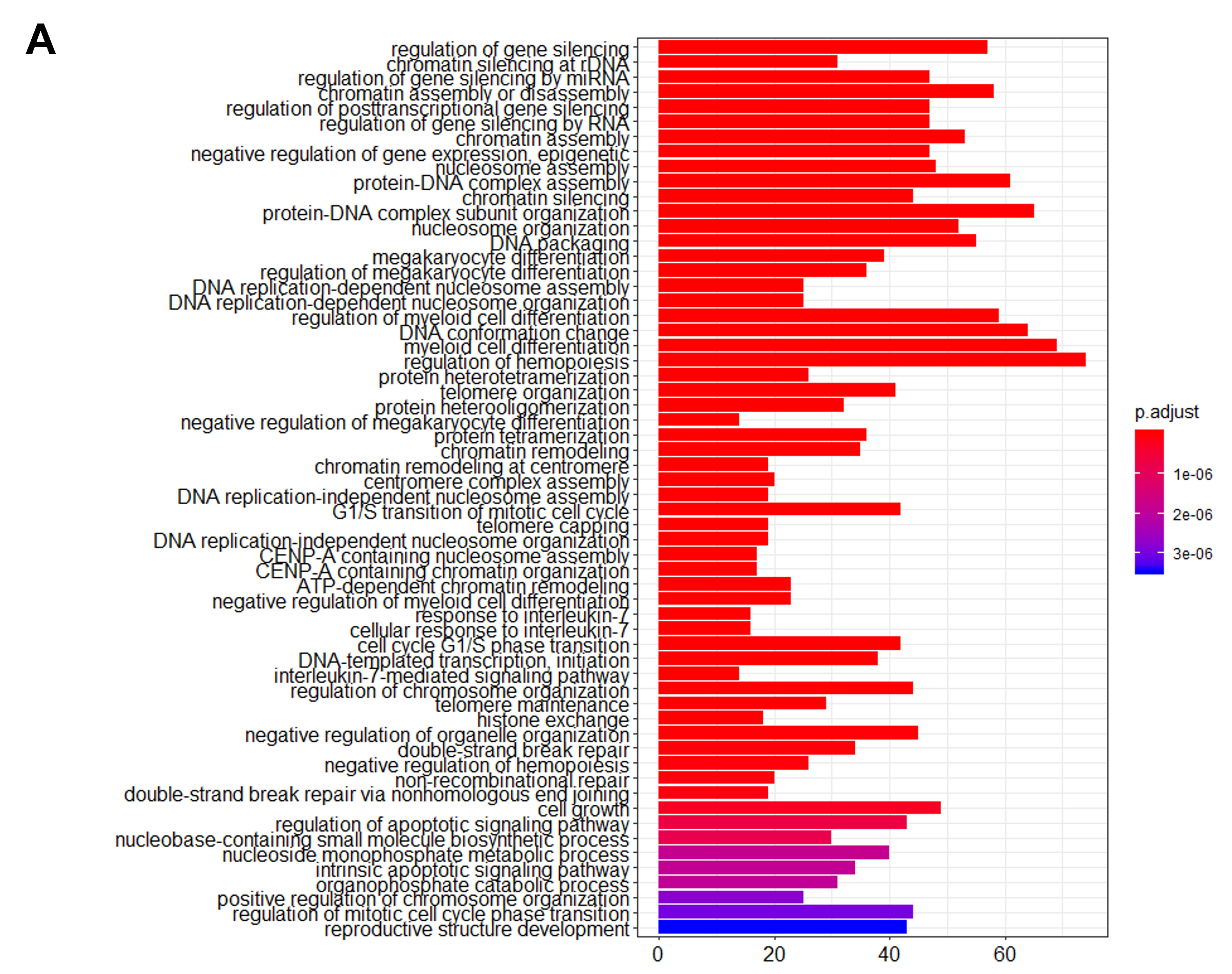

Supplement: Supplementary file 1 [file biomedicines-13-00112-s001.zip › Figure S3A.tif]

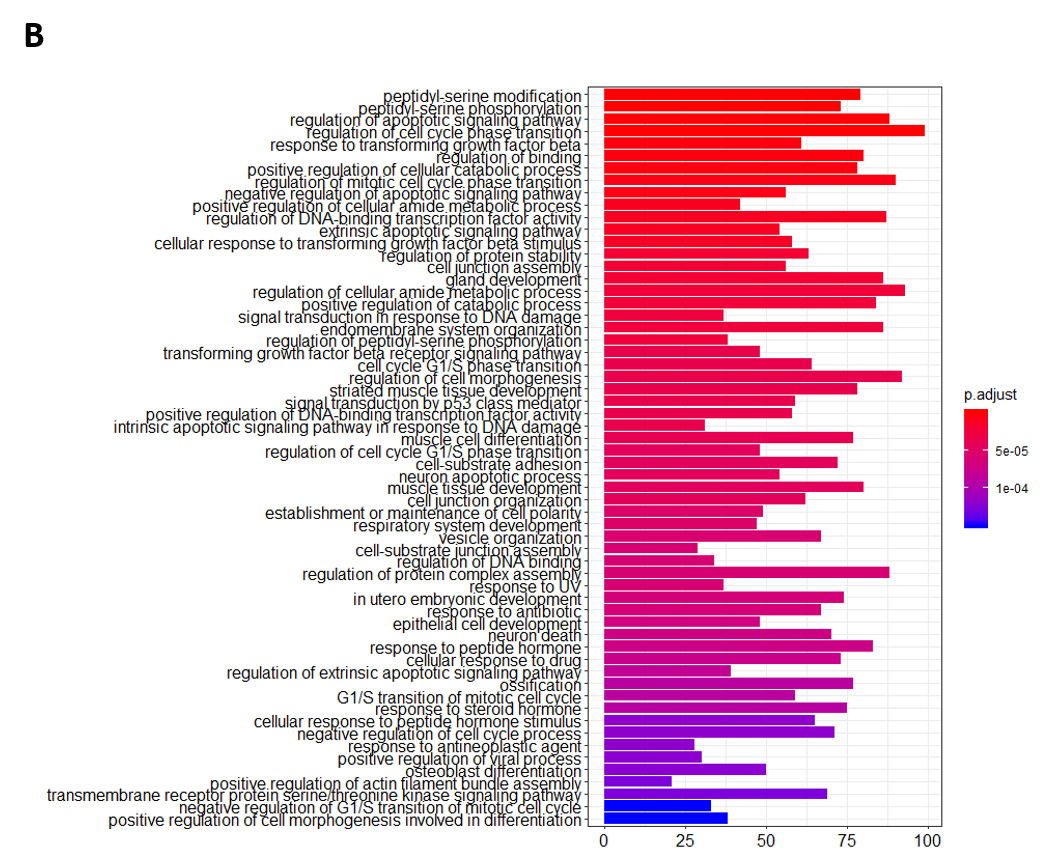

Supplement: Supplementary file 1 [file biomedicines-13-00112-s001.zip › Figure S3B.tif]
